# De novo ablation of premature ventricular complexes originating from posteromedial papillary muscle with pulsed-field energy using a Pentaspline catheter: The first case series in the United States

**DOI:** 10.1016/j.hrcr.2025.04.008

**Published:** 2025-04-17

**Authors:** Mohamed Shokr

**Affiliations:** Department of Electrophysiology, Northernlight Cardiology/Eastern Maine Medical Center, Bangor, Maine

**Keywords:** Papillary muscle, Premature ventricular complex, Pulsed field ablation, Catheter ablation, Pentaspline catheter

**Key Teaching Points**

• Papillary muscle PVCs can be safely and effectively ablated with a pentaspline PFA catheter.
• Continuous intracardiac echocardiography monitoring is key to mitigating risks of entanglement and perforation.
• Further research is needed to determine the least effective number of applications.
• The basket format of the catheter seems to provide the stability that radiofrequency catheters lack in such locations.

## Introduction

Premature ventricular complexes (PVCs) originating from the left ventricular papillary muscles (PMs) account for approximately 4% to 15% of all idiopathic PVCs.^1^ Catheter ablation using radiofrequency energy (RFA) has variable success rates and recurrence risks because of the mobility and intracavitary nature of the PMs, which compromise the catheter stability and lesion durability.^2^^,^^3^ Pulsed-field ablation (PFA) induces cell death through irreversible electroporation via punctuated high-voltage electrical energy fields. PFA achieves rapid, tissue-selective myocardial ablation with minimal collateral damage, a profile validated by multiple trials demonstrating its safety and efficacy in atrial fibrillation (AF) ablation.^4^^,^^5^ Its application to ventricular arrhythmias, however, remains investigational, with early reports suggesting feasibility but limited data on PM-specific outcomes.^6^

We present a case series of de novo PM PVC ablation using a pentaspline PFA catheter (Farapulse, Boston Scientific, Marlborough, MA), exploring its safety and efficacy in this anatomically complex substrate.

## Case 1

**Figure 1:**
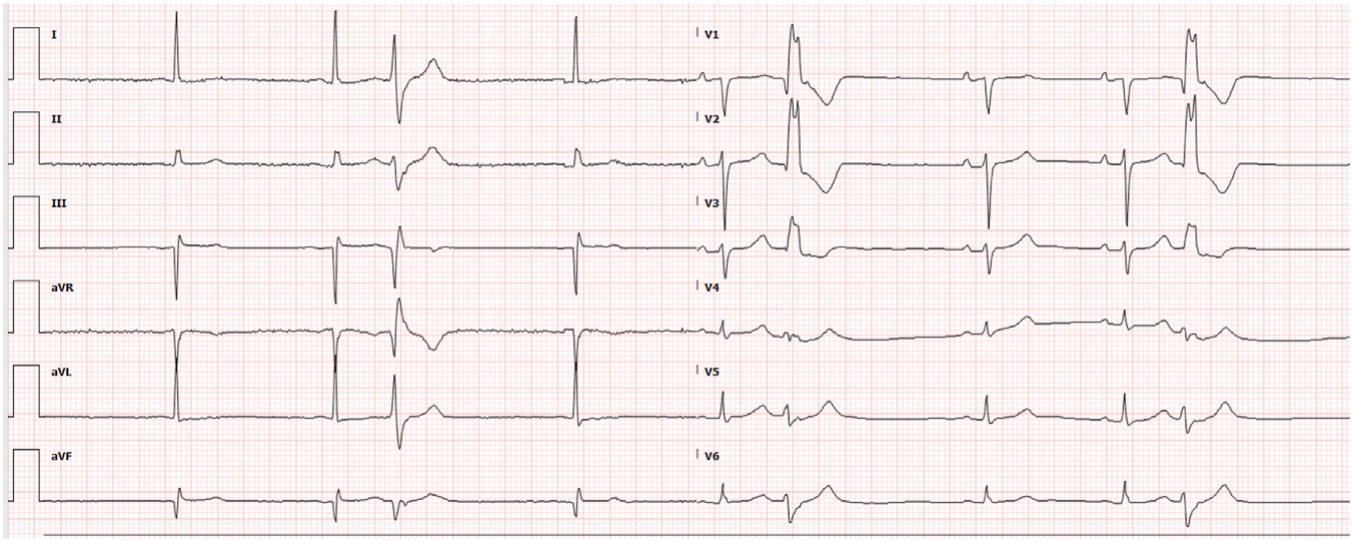
ECG showing the posteromedial papillary muscle PVCs.

A 72-year-old man presented with coronary artery disease (post-coronary artery bypass grafting in 1999, percutaneous coronary intervention to the left circumflex artery in 2013 with chronic total occlusion of obtuse marginal 1, 3 venous grafts, and distal right coronary artery), dyslipidemia, hypertension, cardiomyopathy with an ejection fraction (EF) of 47%, and symptomatic PVCs. A 48-hour Holter monitor showed a 35% PVC burden on metoprolol succinate 50 mg daily. Electrocardiogram (ECG) showed sinus rhythm and monomorphic PVCs consistent with posteromedial PM origin (Figure 1).

**Figure 2:**
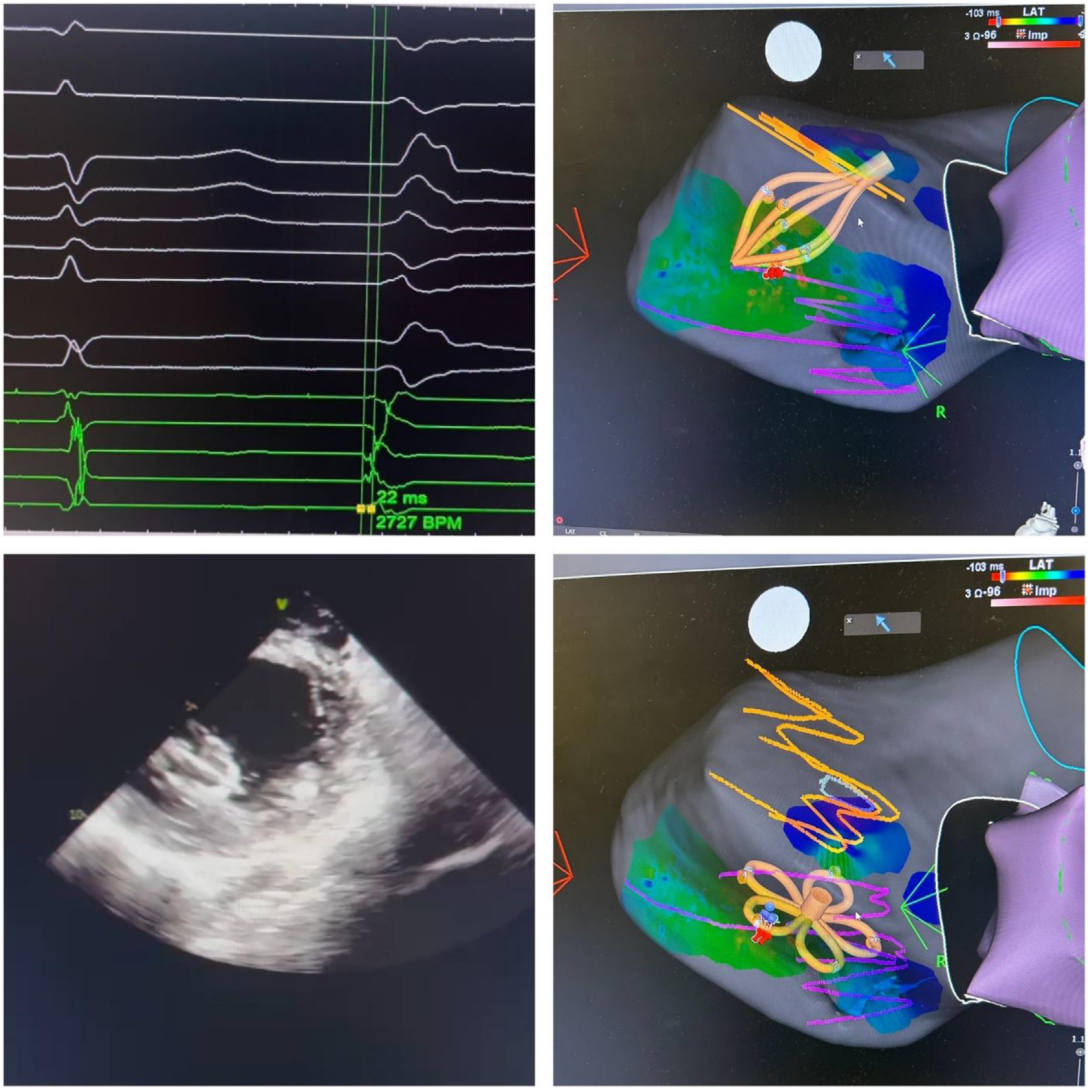
Local electrogram on Farawave catheter 22 ms pre QRS. Farawave catheter in basket configuration sitting on the earliest pre QRS electrogram on the papillary muscle (*red pin*). ICE image of the catheter in basket configuration on top of the PM. The catheter in flower configuration on the papillary muscle.

PVC ablation was performed under general anesthesia. Frequent monomorphic PVCs were induced with isoproterenol (10 μg/min). Right femoral vein access was obtained under ultrasound guidance, with 8F and 8.5F sheaths inserted. Intracardiac echocardiography (ICE) was introduced via the 8.5F sheath, and left ventricular contours were tagged using CARTOSOUND (Biosense Webster, Irvine, CA). The 8F sheath was upsized to an 18F long sheath, facilitating ICE-guided transseptal access with a VersaCross RF wire (Baylis Medical, Montreal, Canada) through a Faradrive sheath (Boston Scientific Inc). Heparin boluses maintained an activated clotting time (ACT) >350 seconds. Activation mapping with an OPTRELL catheter (Biosense Webster) identified the earliest pre-QRS electrogram (16 ms pre-QRS) at the base of the posteromedial PM, with a QS unipolar signal and 96% pace similarity software module (PASO) match. The Farawave PFA catheter (Boston Scientific) was advanced over a guidewire (InQwire , MERITMEDICAL, South Jordan, UT) with a J-shaped atraumatic tip and positioned in a basket configuration at the target site under continuous ICE guidance. The Farawave catheter was registered as a 12F catheter with 20 electrodes and inter-electrode distance of 0.8 mm, and the 0.035-inch J-wire was connected via 2 alligator clips and registered as a 2-mm bipolar catheter. Magnetic field data were acquired using the Optrell catheter, enabling the visualization of the Farawave catheter and the 0.035-inch J-wire with the CARTO3 system. PASO module mapping (Biosense Webster) correlation was 96%, and local electrogram was 22 ms pre QRS signals. PFA energy delivery resulted in immediate PVC suppression. Twelve applications were administered to the surrounding PM area, including 3 in a flower configuration, with no irritative firing observed (Figure 2). No recurrence was noted after a 45-minute waiting period. The patient was discharged 4 hours postprocedure on apixaban (5 mg twice daily) for 2 weeks . A 1-week event monitor at 2 weeks postprocedure showed a PVC burden of 1.8%.

## Case 2

**Figure 3:**
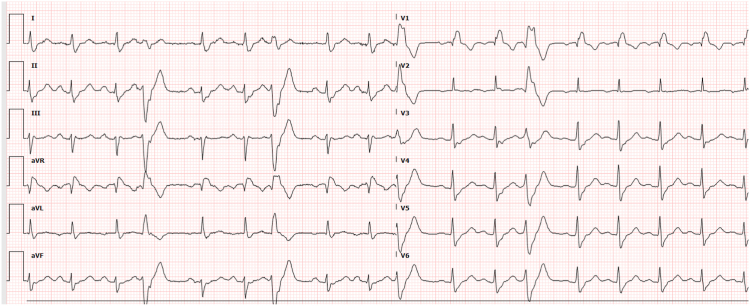
12-lead electrocardiogram (ECG) showing the premature ventricular complexes (PVCs).

A 76-year-old man presented with hypertension, coronary artery disease (post-percutaneous coronary intervention to the left anterior descending and right coronary arteries), obstructive sleep apnea, chronic hypomagnesemia, and symptomatic PVCs . An event monitor showed an 11% PVC burden on diltiazem 240 mg daily. ECG showed right bundle branch block and monomorphic PVCs consistent with posteromedial PM origin (Figure 3). Echocardiography confirmed a normal EF.

PVC ablation was performed under general anesthesia. Frequent monomorphic PVCs were induced with isoproterenol (20 μg/min). Right femoral vein access was obtained under ultrasound guidance, with insertion of 8F and 8.5F sheaths. ICE was introduced via the 8.5F sheath, and left ventricular contours were tagged using CARTOSOUND (Biosense Webster). The 8F sheath was upsized to an 18F long sheath, enabling ICE-guided transseptal access with a VersaCross RF wire (Baylis Medical) through a Faradrive sheath (Boston Scientific). Heparin boluses maintained an ACT >350 seconds.

**Figure 4:**
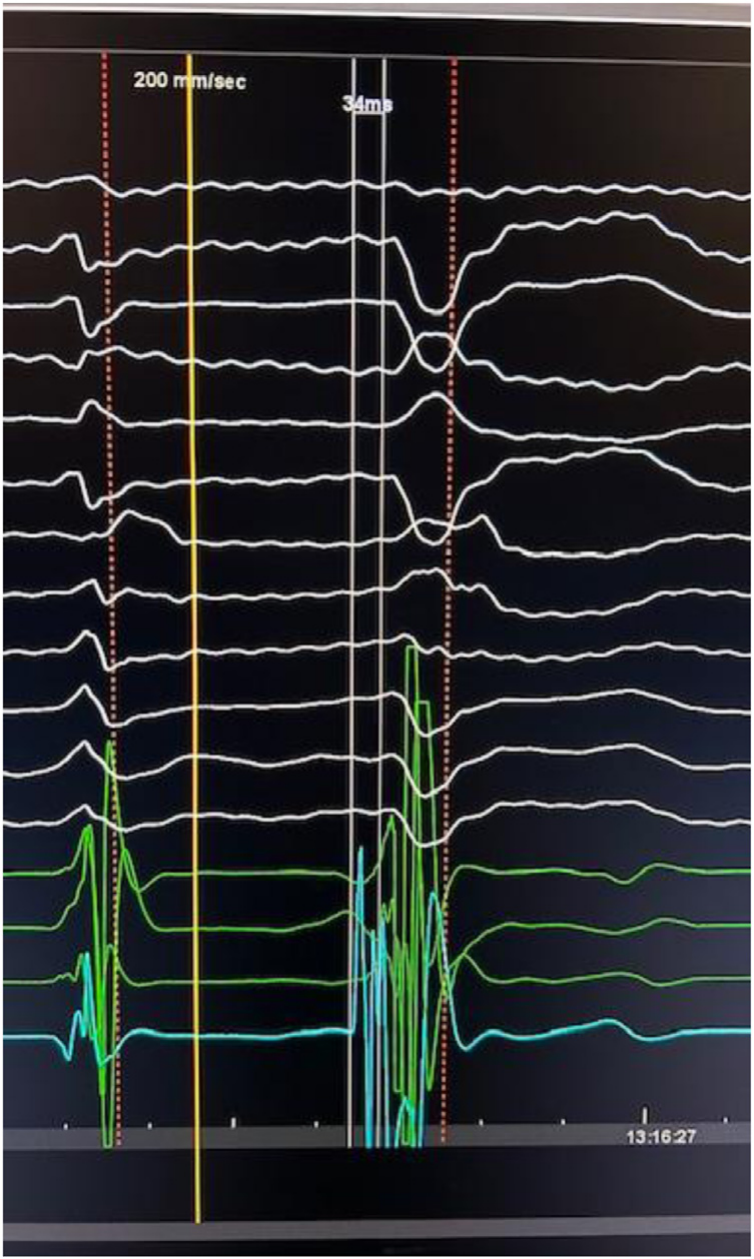
Local electrogram on Farapulse catheter 34 ms pre QRS.

Activation mapping with an OPTRELL catheter (Biosense Webster) identified the earliest pre-QRS electrogram (20 ms pre-QRS) at the base of the posteromedial PM, with a QS unipolar signal and 96% PASO match. The Farawave PFA catheter (Boston Scientific) was advanced over a wire and positioned in a basket configuration at the target site under continuous ICE guidance. PASO correlation was 88%, with signals 34 ms pre-QRS. PFA energy delivery achieved immediate PVC suppression. Three applications were administered to the surrounding PM area, with irritative ventricular firing noted during each application (Figure 4).

No recurrence occurred after a 45-minute waiting period. The patient was discharged 4 hours postprocedure on apixaban (5 mg twice daily) for 2 weeks, with diltiazem discontinued. A 1-week event monitor at 6 weeks postprocedure showed a PVC burden of <1%.

## Case 3

**Figure 5:**
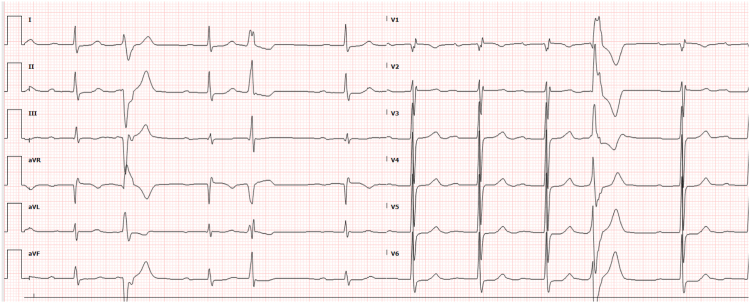
12-Lead electrocardiogram (ECG) showing the papillary muscle (PM) premature ventricular complexes (PVCs).

An 83-year-old man had a history of hypertension, lung cancer (post-immunotherapy), adrenal insufficiency, pulmonary hypertension, and symptomatic PVCs. Holter monitoring indicated a PVC burden of 44% on metoprolol succinate 25 daily. ECG demonstrated sinus rhythm with first-degree atrioventricular delay and monomorphic PVCs suggestive of posteromedial PM origin (Figure 5). Echocardiography confirmed a normal EF, and cardiac magnetic resonance imaging showed no late gadolinium enhancement.

PVC ablation was pursued under general anesthesia. Frequent monomorphic PVCs were induced with isoproterenol (10 μg/min). Right femoral vein access was obtained under ultrasound guidance, and 8F and 8.5F sheaths were inserted. ICE was introduced via the 8.5F sheath, with left ventricular contours tagged using CARTOSOUND (Biosense Webster). The 8F sheath was upsized to an 18F long sheath, facilitating ICE-guided transseptal access with a VersaCross RF wire (Baylis Medical) through a Faradrive sheath (Boston Scientific). Heparin boluses maintained an ACT >350 seconds.

**Figure 6:**
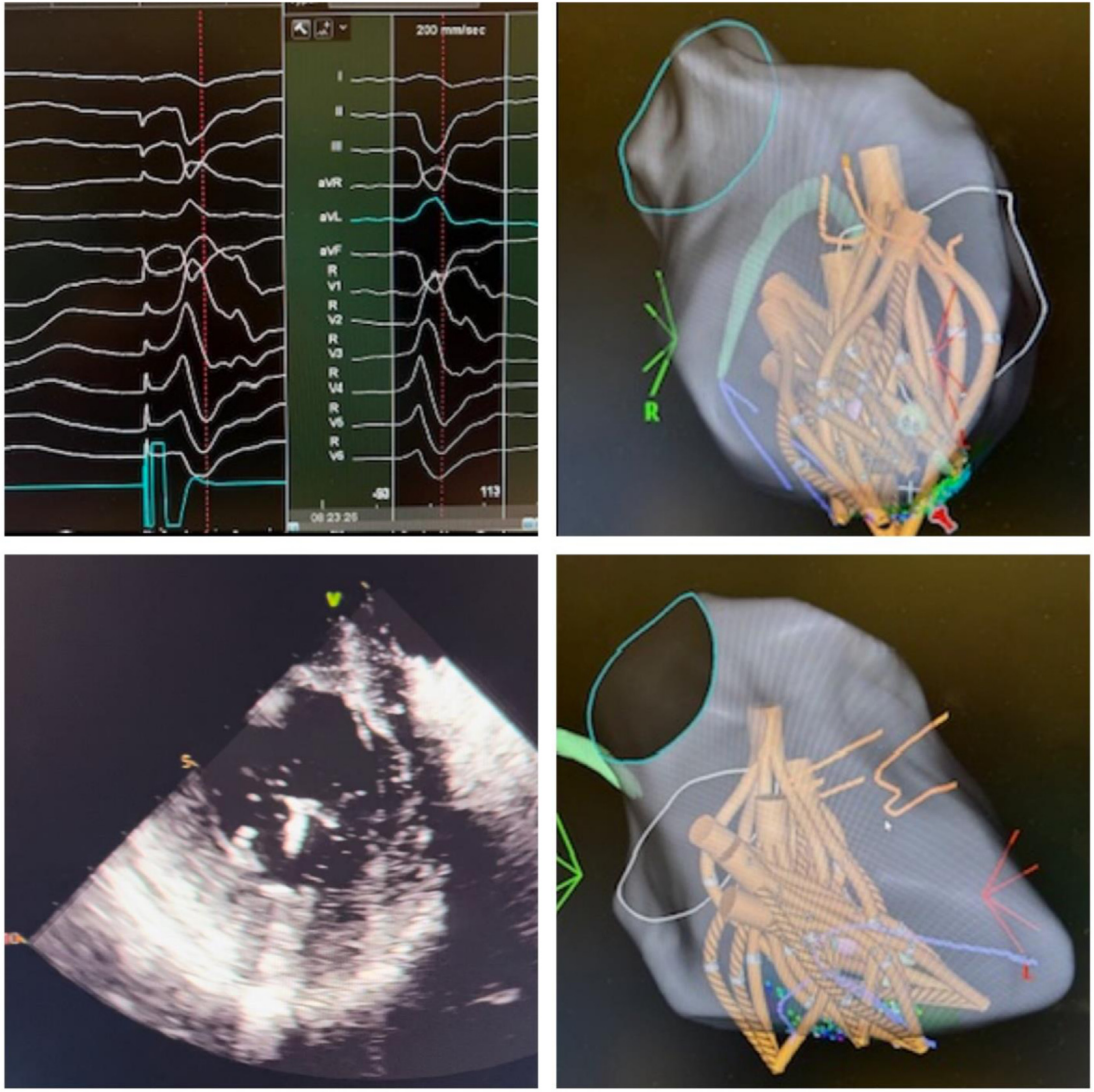
Pace mapping through the FARWAVE catheter. The Farawave catheter in basket configuration on the papillary muscle in left anterior oblique and right anterior oblique views. Intracardiac echocardiography image of the catheter on the papillary muscle.

Activation mapping with an OPTRELL catheter (Biosense Webster) identified the earliest pre-QRS local electrogram (27 ms pre-QRS) at the posteromedial PM tip, with a PASO match of 98% and a QS unipolar signal. The Farawave PFA catheter (Boston Scientific) was advanced over a wire and positioned in a basket configuration at the target site under continuous ICE guidance. PASO correlation was 97% via the Farawave catheter (Figure 6). PFA energy delivery resulted in immediate PVC suppression. Twelve applications were administered across the surrounding PM region in basket configuration, with transient irritative ventricular firing

No recurrence was noted after a 45-minute waiting period. The patient was discharged 4 hours postprocedure on apixaban (5 mg twice daily) for 2 weeks. A 1-week event monitor at 4 weeks postprocedure indicated a PVC burden of <1%.

## Discussion

Ablation of PM PVCs poses significant challenges because of catheter instability and PM thickness, contributing to lower success rates with RFA.^7^, ^8^, ^9^ PFA, a nonthermal modality with proven safety and efficacy in atrial fibrillation ablation,^10^ is emerging as a promising alternative for ventricular arrhythmias, although its application to PM PVCs remains underexplored.

Preclinical studies support PFA’s potential in this context. In a porcine model, a lattice-tip PFA catheter achieved 6-mm lesion depth with 3 to 5 applications at PM sites, with homogeneous fibrosis observed at 21 days and no mitral valve damage on ICE.^10^ Additional preclinical data suggest that PFA preserves arterioles and nerve fascicles, potentially reducing collateral injury.^11^ Clinical reports of PFA for PM PVCs are limited, with few cases describing its use after RFA failure^12^^,^^13^ or via alternative approaches (eg, retrograde aortic).^14^ Recent series using focal PFA (CENTAURI generator) reported acute success in 1 of 2 posteromedial PM PVC cases and long-term PVC burden reduction (≥80%) in both, although ventricular irritative firing occurred in approximately half of their patients.^15^ Initial experiences with the FARAPULSE PFA system for ventricular arrhythmias also show promise.^14^^,^^16^, ^17^, ^18^

In our series of 3 patients with de novo PM PVCs treated using the Farawave pentaspline PFA catheter after obtaining the consent for the off-label use, we achieved acute suppression in all cases and significant PVC burden reduction (<1% to 1.8%) at follow-up (2–6 weeks). The basket configuration likely enhanced stability compared with RFA, addressing a key limitation at this dynamic intracavitary site. Continuous ICE monitoring proved essential, mitigating risks of catheter entrapment and mitral valve injury (postprocedure echocardiography showed no regurgitation). Positioning the catheter on the cavitary PM surface may minimize unnecessary ablation of the left ventricular wall, optimizing lesion specificity. We advanced the catheter over a guidewire with an atraumatic tip to mitigate risks of perforation. We observed irritative ventricular firing in 2 of 3 cases, less frequently than reported elsewhere,^15^ possibly because of differences in catheter design or energy delivery.

In swine models, the Pentaspline catheter achieved lesion depth up to 5.7 mm in the right ventricular outflow tract,^19^ and an investigational focal contact force-sensing PFA ablation catheter (modified IntellaNav StablePoint, Boston Scientific) achieved an average depth of 8.1 mm (compared with 4.5 mm with RF) at the papillary muscle with 1 lesion. This highlights the impact of the catheter footprint on the lesion depth.^20^

Compared with RFA, PFA offered, in our experience, shorter procedure times and both acute and sustained efficacy. The variable PFA applications^3^, ^4^, ^5^, ^6^, ^7^, ^8^, ^9^, ^10^, ^11^, ^12^ and configurations suggest adaptability to PM anatomy, warranting further study. Our findings align with growing evidence^13^, ^14^, ^15^, ^16^, ^17^, ^18^ that PFA is a safe, effective option for PM PVCs, potentially surpassing RFA in stability and efficiency while maintaining a favorable safety profile.

## Conclusion

Our case series demonstrates that PFA using the pentaspline Farawave catheter is a safe and effective approach for ablating PM PVCs. PFA achieved acute suppression in all cases and reduced PVC burden to <1% to 1.8% at 2–6 weeks postprocedure, with no mitral valve complications observed on echocardiography. The basket configuration enhanced catheter stability, and continuous ICE monitoring ensured procedural safety. These findings suggest PFA may overcome limitations of RF ablation in this challenging substrate. However, larger, prospective studies with extended follow-up are needed to confirm long-term efficacy and define optimal PFA strategies for PM PVCs.

## Disclosures

The authors have no conflicts of interest to disclose.
